# Murine FLT3 ligand-derived dendritic cell-mediated early immune responses are critical to controlling cell-free human T cell leukemia virus type 1 infection

**DOI:** 10.1186/1742-4690-11-S1-P70

**Published:** 2014-01-07

**Authors:** Saifur Rahman, Zafar K Khan, Brian Wigdahl, Stephen Jennings, Frederic Tangy, Pooja Jain

**Affiliations:** 1 Drexel Institute for Biotechnology and Virology Research, and the Department of Microbiology and Immunology, Drexel University College of Medicine, Doylestown, PA, USA

## 

Human T cell leukemia virus type 1 (HTLV-1) is associated with two immunologically distinct diseases: HTLV-1-associated myelopathy/tropical spastic paraparesis and adult T cell leukemia. We observed previously that depletion of dendritic cells (DCs) in CD11c-diphtheria toxin receptor transgenic mice followed by infection with cell-free virus led to greater proviral and Tax mRNA loads and diminished cellular immune response compared with mice infected with cell-associated virus. To understand the significance of these in vivo results and explore the host-pathogen interaction between DCs and cell-free HTLV-1, we used FLT3 ligand-cultured mouse bone marrow-derived DCs (FL-DCs) and chimeric HTLV-1. Phenotypically, the FL-DCs upregulated expression of surface markers (CD80, CD86, and MHC class II) on infection, however, the level of MHC class I remained unchanged. We performed kinetic studies to understand viral entry, proviral integration, and expression of the viral protein Tax. Multiplex cytokine profiling revealed production of an array of proinflammatory cytokines and type 1 IFN (IFN-α) by FL-DCs treated with virus. Virus-matured FL-DCs stimulated proliferation of autologous CD3(+) T cells as shown by intracellular nuclear Ki67 staining and produced IFN-γ when cultured with infected FL-DCs. Gene expression studies using type 1 IFN-specific and DC-specific arrays revealed upregulation of IFN-stimulated genes, most cytokines, and transcription factors, but a distinct downregulation of many chemokines. Overall, these results highlight the critical early responses generated by FL-DCs on challenge with cell-free chimeric HTLV-1.

